# Diagnostic Performance of Red Blood Cell Indices in the Differential Diagnosis of Iron Deficiency Anemia and the Thalassemia Trait in Chile: A Retrospective Study

**DOI:** 10.3390/diagnostics14212353

**Published:** 2024-10-22

**Authors:** Mario Balcázar-Villarroel, Angélica Mancilla-Uribe, Sandra Navia-León, Florencia Carmine, Katherine Birditt, Cristian Sandoval

**Affiliations:** 1 Laboratorio Clínico TecnoMedic, Puerto Montt 5502901, Chile; informaciones.tecnomedic@gmail.com (A.M.-U.); sandra.navia@gmail.com (S.N.-L.); 2 Carrera de Medicina, Facultad de Medicina, Universidad de La Frontera, Temuco 4811230, Chile; f.carmine02@ufromail.cl; 3 Physiology Development and Neuroscience Department, University of Cambridge, Cambridge CB2 1TN, UK; krb56@cam.ac.uk; 4 Escuela de Tecnología Médica, Facultad de Salud, Universidad Santo Tomás, Los Carreras 753, Osorno 5310431, Chile; 5 Departamento de Medicina Interna, Facultad de Medicina, Universidad de La Frontera, Temuco 4811230, Chile; 6 Núcleo Científico y Tecnológico en Biorecursos (BIOREN), Universidad de La Frontera, Temuco 4811230, Chile

**Keywords:** β-thalassemia trait, differential diagnosis, iron deficiency anemia, RBC indices

## Abstract

Background: Iron deficiency anemia (IDA) and the β-thalassemia trait (BTT) are two main causes of hypochromic–microcytic anemia worldwide. Researchers have described many red blood cell (RBC) indices as screening tests for presumptive differentiation, based on differences observed in complete blood count (CBC) data for each condition. There are few BTT reports in Chile, and neither laboratories nor clinical staff have widely used these indices. Objective: The objective of this study was to evaluate the diagnostic performance of 29 RBC indices in 182 patients (51 BTT and 131 IDA) and compare CBC results in both groups. Methods: A retrospective search was carried out in the Laboratory Information System between January 2021 and February 2024 to collect results from CBC, and 29 RBC indices were calculated for each patient. Then, sensitivity, specificity, positive predictive value, negative predictive value, Youden’s index, positive likelihood ratio, negative likelihood ratio, and diagnostic accuracy were calculated using MedCalc©. Results: The Green and King, Wongprachum, and Keikhaei indices showed the best discriminatory power with Youden index values of 0.923, 0.908, and 0.896, respectively, and significant differences were observed in all CBC parameters between BTT and IDA patients (*p* < 0.001). Conclusions: The Green and King, Wongprachum, and Keikhaei indices showed the best performance; therefore, they can be used as screening for the differential diagnosis between BTT and IDA in order to improve diagnosis given the important therapeutic and epidemiological implications. In this way, clinical laboratories could have a main role in the investigation of these patients.

## 1. Introduction

The World Health Organization (WHO) defines anemia as a condition in which the number of red blood cells or the total hemoglobin (HB) concentration within them is lower than normal in the blood. The diagnosis cut-off point varies based on sex, age, and physiological condition, impacting an estimated 1.62 billion people worldwide, with a higher prevalence in developing countries compared with developed ones [1,2]. The most common way to group morphological anemias is by their Mean Corpuscular Volume (MCV) in microcytic, normocytic, and macrocytic [3]. Among microcytic anemias, thalassemias and iron deficiency anemia (IDA) are the most frequent causes worldwide. In β-thalassemia, there is a partial or total decrease in the synthesis of β-globin chains in HB due to genetic mutations [4]. Currently, the following conditions are recognized: β-thalassemia trait (BTT), β-thalassemia intermedia (BTI), and β-thalassemia major (BTM) [4]. BTT is caused by a heterozygous mutation and has no symptoms. It can be identified by its specific hematological features [5,6], which are confirmed by HB electrophoresis (HbA2 fraction > 3.5%) [7,8]. On the other hand, IDA is the cause of approximately 50% of anemias worldwide and mainly affects children and women of childbearing age [1,9]. Although both anemias are of the microcytic type, BTT has a greater degree of decrease in MCV, a smaller decrease in HB, and a higher erythrocyte count [9,10,11]. Furthermore, studies have demonstrated a greater prevalence of basophilic stippling in BTT compared with IDA [9,12,13]. Based on these findings, researchers have described several discriminatory RBC indices to differentiate between BTT and IDA presumptively. These indices can serve as screening tests for more specific and expensive studies that determine the cause of anemia [14,15,16,17,18,19,20,21,22,23,24,25,26,27,28,29,30,31,32,33,34,35,36,37,38,39].

In Chile, specialist doctors at the tertiary level typically diagnose BTT, but few studies present a series of diagnosed cases or evaluate the performance of discriminatory RBC indices described in the specialized literature [40]. Furthermore, the majority of the limited published studies focus on isolated cases of BTT and/or BTM in pregnant patients and/or their offspring [41,42,43], which means the discriminatory power of these RBC indices has not been estimated in our population.

There are various equations for differentiating BTT from IDA. Each of these differentiators possesses unique sensitivity, specificity, and Youden indices, leading to a variety of reported results [33,37,44,45]. The present study is innovative because it compares 29 differential equations simultaneously. Many related research studies used fewer equations than our study to diagnose BTT from IDA. Furthermore, this study was the first in the region. Therefore, given this lack of information at the national level, the present study aimed to evaluate the ability of 29 differential equations consisting of simple blood parameters to separate individuals with BTT from those with IDA by calculating sensitivity, specificity, positive predictive value, negative predictive value, Youden’s index, positive likelihood ratio, negative likelihood ratio, and diagnostic accuracy. Therefore, the two objectives of this study were to evaluate the diagnostic performance of 29 RBC indices in patients diagnosed with BTT and IDA and to compare complete blood count (CBC) values between both groups of patients.

## 2. Materials and Methods

A retrospective search was carried out in the Laboratory Information System (LIS, ProActive©, Christchurch, New Zealand) between January 2021 and February 2024 in order to collect results from the following determinations of patients with IDA in Puerto Montt (Chile), confirmed using iron deficiency anemia as diagnostic criteria or BTT confirmed using HB electrophoresis.

### 2.1. CBC

Samples were processed using the Mindray BC-5380 (Mindray Medical International Limited, Shenzhen, China) analyzer. It was performed in IDA and BTT patients.

### 2.2. Iron Metabolism

Samples were processed using the Mindray BS-480 (Mindray Medical International Limited, Shenzhen, China) analyzer. It was performed only in IDA patients.

### 2.3. Ferritin

A MAGLUMI-800 (Snibe Diagnostic^®^, Shenzhen, China) analyzer processed the ferritin samples. It was performed only in IDA patients.

### 2.4. Hemoglobin Electrophoresis

Red-Lab Laboratories (Santiago de Chile, Chile) processed the samples for hemoglobin electrophoresis at an alkaline pH. It was performed only for confirmation in BTT patients.

### 2.5. Morphological Characteristics

Peripheral blood smears were stained with May Grünwald–Giemsa (Merck, Darmstadt, Germany) and observed at 100× magnification. It was performed in IDA and BTT patients.

### 2.6. Diagnostic Criteria

For the inclusion of patients in each anemia group, the following diagnostic criteria were used:

#### 2.6.1. IDA

In adult men: HB < 13 g/dL and ferritin < 28 ng/mL; or HB < 13 g/dL and iron blood < 65 ug/dL plus transferrin saturation (TS) < 16%. In adult women: HB < 12 g/dL and ferritin < 12 ng/mL; or HB < 12 g/dL and iron blood < 50 ug/dL plus TS < 16%. In children (<15 years): HB < RV (reference value) according to age and ferritin <12 ng/mL; or HB < RV according to age and iron blood < 30 ug/dL plus TS < 16% [44,46].

##### 2.6.2. β-Thalassemia Trait

Children (> 1 year) and adults (men and women): HB electrophoresis at alkaline pH with HbA fraction <96.5% and HbA2 >3.5% [8].

### 2.7. Exclusion Criteria

The patients with HB concentrations less than 9.0 g/dL were excluded because it is assumed that IDA is the underlying cause in these cases and did not consider differential diagnosis a problem [44].

### 2.8. Calculation of Discriminatory RBC Indices

The CBC data of patients in each group was tabulated in Excel for Mac (version 16.89.1, Microsoft Office). The discriminatory RBC indices shown in Table 1 were calculated using the originally published cut-off points [14,15,16,17,18,19,20,21,22,23,24,25,26,27,28,29,30,31,32,33,34,35,36,37,38,39].

### 2.9. Statistical Analysis

The performance of each index was evaluated using the following parameters: sensitivity (Se), specificity (Sp), positive predictive value (PPV), negative predictive value (NPV), Youden’s index, positive likelihood ratio (PLR), negative likelihood ratio (NLR), and diagnostic accuracy. These parameters were obtained from the contingency table, which elaborated each index according to the following scheme [47,48,49]:

**ΒTT (+)****BTT (−)/(IDA)****Total**RBC indices suggest BTT(A) True Positive(B) False PositivePositivesRBC index does not suggest BTT(C) False Negative(D) True NegativeNegatives
Sick patientsHealthy patientsNBTT: β-thalassemia trait; IDA: iron deficiency anemia; RBC: red blood cell count.


Patients in Box “A” had a discriminatory RBC index that suggested the presence of BTT and met the diagnostic criteria for BTT. Box “B” encompassed patients whose discriminatory RBC index evaluation indicated the presence of BTT and who satisfied the IDA diagnostic criteria. Box “C” encompassed patients where the evaluation of a discriminatory RBC index indicated the presence of IDA, and they satisfied the BTT diagnostic criteria. Patients in Box “D” had a discriminatory RBC index evaluation suggesting the presence of IDA and meeting IDA diagnostic criteria. Table 2 shows the confusion matrix of discriminatory RBC indices.

The likelihood ratio was estimated based on sensitivity and specificity parameters as follows:

$$PLR=\frac{True\;positive\;rate}{False\;positive\;rate}=\frac{Sensitivity}{1-Specificity}$$

$$NLR=\frac{False\;negative\;rate}{True\;negative\;rate}=\frac{1-Sensitivity}{Specificity}$$

Table 3 reflects the impact of PLR and NLR values. In general, a PLR greater than 10 and an NLR less than 0.1 signify a significant change in the pretest probability, thereby determining a change in clinical behavior with high certainty [50,51].

The online software MedCalc^®^ (https://www.medcalc.org/, accessed on 22 September 2024) was used to calculate the aforementioned parameters (with their respective confidence intervals) and compare two proportions in the presence or absence of basophilic stippling (chi-square test). Also, the online software GraphPad (https://www.graphpad.com, accessed on 22 September 2024) was used to determine statistically significant differences in age and CBC parameters between both groups (Student’s *t*).

## 3. Results

A search in LIS permitted the inclusion of 182 patients who met the previously defined diagnostic criteria (131 in the IDA group and 51 in the BTT group). The age range for BTT patients was between 1 and 95 years (mean: 44.3 and SD: 23.4), and for IDA patients, it was between 12 and 90 years (mean: 43.0 and SD: 17.6) (statistically non-significant differences).

Table 4 displays the performance of the discriminatory RBC indices. None of them showed 100% accuracy. In this sense, some indices had high sensitivity and very low specificity, such as the Kandhro 1, Chandra, Das Gupta, and Shine and Lal indices. In contrast, some indices had high specificity but very low sensitivity, such as the Sirachainan, Telmissani MDHL, and England and Fraser I indices. Youden’s index, a diagnostic accuracy measure based on sensitivity and specificity (with a minimum value of −1 and a maximum value of +1) [52], revealed that the Green and King index had the highest Youden’s index value, followed by the Wongprachum and Keikhaei indices. The RBC indices with the lowest values were the Telmissani MCHD, Kandhro 1, and Bessman indices.

It has been shown that a diagnostic test has a high level of usefulness when PLR is greater than 10 and NLR is less than 0.10 [53]. LR values are the ratio between the chance of a result happening in people who have the disease and the chance of that result happening in people who do not have the disease. The Green and King, Wongprachum, and Keikhaei RBC indices met this criterion. According to diagnostic accuracy, defined as the probability that a test result correctly predicts the presence or absence of disease (with a minimum value of 0% and a maximum value of 100%) [49], the Green and King, Matos and Carvalho, and Sirdah indices had the highest values among the RBC indices. On the other hand, the Telmissani MCHD, Kandhro 1, and Das Gupta indices had the lowest values.

Table 5 shows the CBC results for both groups. The BTT group had a higher RBC count, higher HB, lower MCV, lower MCH, higher MCHC, lower platelet count (PQ), higher mean platelet volume (MPV), and lower red-cell distribution width (RDW) compared with the IDA group (*p* < 0.05). Furthermore, a greater presence of basophilic stippling in BTT patients (90.2%) compared with IDA patients (0.0%) was observed (*p* < 0.001).

## 4. Discussion

Microcytosis and hypochromia are prevalent manifestations of both IDA and βTT. The morphological characteristics in both IDA and βTT are occasionally so similar that distinguishing between them is somewhat challenging [54]. The distinction between βTT and IDA can be efficiently achieved using a series of assays, including serum ferritin, serum iron, and HbA2 level assessment [55].

The differentiation between βTT and IDA requires HbA2 estimation by Hb electrophoresis, examination of a peripheral blood film, serum ferritin, iron, TIBC, and transferrin saturation. However, because these methods are relatively expensive and time-consuming, it is preferable to rely on simple and already available information. However, automated analyzers provide RDW in CBCs, which can be associated with a derived value, the red-cell distribution width index, to distinguish IDA and BTT [56].

Sensitivity and specificity are critical test accuracy metrics that allow healthcare providers to assess the suitability of the tests. It is important for providers to use diagnostic tests that give them enough confidence based on their sensitivities, specificities, positive predictive values (PPVs), negative predictive values (NPVs), positive likelihood ratios (PLRs), and negative likelihood ratios (NLRs) [57]. Sensitivity is the ratio of genuine positive tests to the total number of patients with a condition, meaning that a test or device’s ability to yield a positive result for a person with the condition is crucial [58]. On the other hand, specificity refers to the proportion of true negatives among all individuals who do not possess an illness or condition [58], meaning that the test or instrument’s ability to produce a normal range or negative results for an individual without the condition is important. The relationship between sensitivity and specificity is inverse; an increase in sensitivity usually leads to a decrease in specificity, and vice versa [59,60].

The PPVs ascertain the proportion of genuine positives among all positive findings, whereas NPVs ascertain the proportion of true negatives among all negative findings. As the value ascends toward 100, it nears a gold standard [59].

LRs serve as an additional statistical instrument for comprehending diagnostic testing. LRs enable clinicians to assess the extent to which the use of a specific test will modify the probability. A PLR is defined as the probability of obtaining a positive test result in a patient with the disease divided by the probability of obtaining a positive test result in a patient without the condition, while an NLR is defined as the probability that a patient with a disease test negative divided by the probability that a patient without the condition tests negative [58].

Our study is one of the first of its kind conducted in Chile, and we were able to demonstrate the good performance of several discriminatory indices, such as the Green and King, Wongprachum, and Keikhaei indices (Table 4). In addition, significant differences were found in CBC between BTT and IDA patients (Table 5). The results showed a lot of variability in the performance of discriminatory RBC indices. In this sense, the following indices showed high sensitivity but poor specificity: the Shine and Lal, Chandra, Kandhro 1, and Das Gupta indices. Other studies have highlighted this situation with the first index; Batebi et al. [61] reported 96.8% sensitivity and 7.0% specificity, while Jahangiri et al. [39] reported 100% and 17.6%, respectively. Regarding the Chandra index, our work disagrees with its primary evaluation, which determined a sensitivity of 77.1% and a specificity of 73.3% [36], but coincides with the study by Hoffman and Urrechaga [62], who determined that Youden’s index is similar to ours (0.461 vs. 0.359, respectively). The sensitivity and specificity of the Das Gupta index were similar to those found in our study (95.3% vs. 100.0% and 36.2% vs. 35.1%, respectively) [39]. The Kandhro 1 index showed Youden’s index similar to what Sadagheyani et al. [63] found (0.232 vs. 0.243), but not the same as what Laengsri et al. [64] found (0.590). In the opposite direction, some indices showed high specificity but poor sensitivity, as demonstrated by the Sirachainan, Telmissani-MDHL, and England and Fraser I indices. This partially aligns with the findings reported by Ayman et al. [65], who found similar sensitivity and specificity (50.0% and 84.4%, respectively) for the Telmissani MDHL index but very poor sensitivity (0.0%) and high specificity (100.0%) for the Sirachainan index. Regarding the England and Fraser index, our results coincide with those reported by Hoffmann et al. [66], who reported a sensitivity of 75.0% and a specificity of 92.0%.

With respect to good performance indices, the Green and King, Wongprachum, and Keikhaei indices stand out as they demonstrate the best combination of PLR and NLR values and better Youden’s index values (Table 5). Numerous studies have validated the strong performance of these indices, with Green and King receiving the most evaluations [29,39,66]. Similar sensitivity and specificity were found for this index (91.7% and 86.7%, respectively, with an optimized cut-off) in a Spanish population [67]. Our results for the Keikhaei index are the same as its original evaluation, showing similar levels of accuracy (93.4% vs. 93.7%, respectively) [29]. Also, the Wongprachum index performed better in our study than it did in Urrechaga and Hoffmann’s study, which found slightly lower levels of sensitivity and specificity (89.3% and 86.9%, respectively) [67].

At the global level, our results align with several studies that suggest no index has achieved 100% accuracy and that the performance of these indices varies based on the geographical location of the studied population [39,66]. The observed differences between our results and other works could potentially stem from the predominantly adult patient composition in our study, which has demonstrated better performance of discriminatory indices compared with pediatric patients [44,66]. Also, we must consider the exclusion and inclusion criteria employed, the size of the sample, the absence of other thalassemia, and the potential differences in the spectrum of mutations in the Chilean population compared with other nations with more genetically diverse ethnic backgrounds.

In terms of the hematological phenotype seen in both groups of patients (Table 5), our results are the same as those obtained by Chandra et al. [36], who found similar trends in all measured parameters except for MCH, where other studies have found differences [37,63,65]. This situation can be explained by differences in mutations present in each population since some studies show a correlation between the type of mutation (β^0^/β^+^) and some RBC parameters [68].

A correct differentiation between BTT and IDA is essential. First, it avoids iron therapy for patients who do not require it and determines effective treatment with folic acid for patients with BTT who require it (1–5 mg daily). However, this treatment is not essential for patients who consume normal amounts of raw vegetables and/or fruits, except in cases of pregnancy, infections, and major surgeries [69]. Second, it enables genetic counseling for patients with BTT, explaining to the carrier patient that if they have children with someone who has hemoglobinopathy, there is a 25% chance of having offspring with severe clinical conditions and a 50% probability of transmitting BTT to their offspring if the couple has a normal phenotype [69]. Third, the use of discriminatory indices as screening could increase diagnosis in Chile, considering the few studies in the national literature and the lack of reports on the use of these indices in a series of patients [40,41,42,43]. In this case, we suggest that the Green and King, Keikhaei, or Wongprachum index can be reported, along with the appropriate cut-off points, in CBCs that look suspicious, along with a note suggesting that HB electrophoresis be performed for diagnostic confirmation. This proposal relies on the discrete ability of non-specialist physicians to suspect the presence of BTT based on CBC data. In the United States, Hansen et al. [70] demonstrated that 68% of patients suspected of having BTT did not consider this possibility. On the other hand, in Israel, Shalev et al. [71] reported that only 27% of patients with suggestive blood counts had a definitive diagnosis of BTT. Similarly, in India, Kakkar et al. [72] reported that 84% of patients suspected of having BTT did not consider this possibility, and only 7.1% achieved a definitive diagnosis. In Latin America, Ruiz [73] reported that BTT is underdiagnosed and frequently confused with IDA in Mexico.

Another finding revealed a significantly higher occurrence of basophilic stippling in BTT (90.2%) compared with IDA (0.0%) (Table 4), indicating a higher observation rate than previously reported by Aixalá (72.3%) [9], Harrington et al. (16.7%) [12], and Lazarte et al. (67.0%) [13]. Despite the fact that this inclusion can occur in a variety of conditions [74], our experience indicates that it is highly indicative of BTT in microcytic anemia, which rules out rare lead poisoning. A medical technologist should focus their microscopic observation on BTT only after analyzing the CBC results and the patient’s past records.

Despite the aforementioned points, it is crucial to acknowledge the limitations of our research. Given the type of study, it was not possible to determine if any inflammatory or infectious disorders could concurrently cause anemia in any patient group. This is because, in the absence of symptoms in clinics that warrant such research, it becomes challenging to request such tests in an outpatient clinical laboratory for patient cost-effectiveness. Furthermore, because of our study’s retrospective nature and the relatively high cost of hemoglobin electrophoresis in Chile, it was not possible to perform this examination in the IDA patient group, making it impossible to confirm the absence of BTT patients reliably. However, it is important to note that the probability of a patient presenting both conditions (BTT and IDA) is very small given the low prevalence of BTT in our country, estimated at 0.06% of the general population [40]. The RBC indices’ performances would have changed little for the same reason. Chile, for example, lacks access to molecular biology technology for thalassemia, and unlike other countries in the region [75], the mutations present in our population have not been characterized so far.

It is crucial to consider the benefits of using these discriminatory RBC indices, including the fact that they do not add to the cost of CBC, thereby enhancing the cost–benefit ratio of the determination. They also serve as a screening tool, objectively supporting the later realization of more expensive tests like HB electrophoresis. Furthermore, the simplicity of formulas allows them to be widely available in places with few technological resources, given the lack of need to incorporate more complex analyzers [34,44,76].

### Scope and Limitations

Although the RBC indices are similar in terms of parameters used and calculation formula, they have shown differences in performance. In fact, RBC indices have been used to differentiate between BTT and IDA in low- and middle-income countries and low-prevalence contexts prior to performing HB electrophoresis, and therein lies its main clinical application. In the Supplementary Materials, we include an online calculator for calculating RBC indices in a clinical setting (Table S1).

Some limitations of the study are 1. the relatively small cohort of subjects, 2. the impossibility of carrying out molecular biology, and 3. the impossibility of clarifying conclusively whether IDA and BTT could be present at the same time. However, because of the low prevalence in Chile, this situation is very unlikely in a relatively small cohort of patients.

Further research is required to compare the discriminative capabilities of all red cell indices documented in the literature to date; however, the current work offers a chance to employ more straightforward methods for distinguishing between two ambiguous conditions. Furthermore, despite the benefits and ease of implementing blood indices, discriminative formulas have limitations because they are unable to distinguish all instances of IDA from BTT.

## 5. Conclusions

Our research showed that Green and King, Wongprachum, and Keikhaei indices had great performance (with a Youden’s index of 0.923, 0.908, and 0.896, respectively), and, when reported in conjunction with the CBC, can effectively raise clinical suspicion in patients with BTT. On the other hand, significant differences were observed in all CBC parameters between BTT and IDA patients (*p* < 0.001). Future studies with a bigger sample size and in more health centers across the country will be able to confirm these findings. This will make it possible for clinical laboratories in Chile to use them regularly to suggest BTT diagnosis on a large scale.

## Figures and Tables

**Table 1:** Discriminatory RBC indices.

RBC Index	Formula	Cut-Off BTT	Cut-Off IDA
England and Fraser I [14]	MCV − RBC − (5 × HB)	<0.0	>0.0
Srivastava [15]	MCH/RBC	<3.8	>3.8
Mentzer [16]	MCV/RBC	<13	>13
Shine and Lal [17]	MCV × MCV × (MCH/100)	<1530	>1530
England and Fraser II [18]	MCV − RBC − (5 × HB) − 3.4	<0.0	>0.0
Bessman [19]	RDW	<14	>14
Ricerca [20]	RDW/RBC	<3.3	>3.3
Green and King [21]	(MCV × MCV × RDW)/(100 × HB)	<65	>65
Das Gupta [22]	(1.89 × RBC) − (0.33 × RDW) − 3.28	>0.0	<0.0
Jayabose-RDW [23]	(MCV × RDW)/RBC	<220	>220
Telmissani MCHD [24]	MCH/MCV	<0.34	>0.34
Telmissani MDHL [24]	(MCH × RBC)/MCV	>1.75	<1.75
Huber–Herklotz [25]	(MCH × RDW/10 × RBC) + RDW	<23	>23
Kerman I [26]	(MCV × MCH)/RBC	<300	>300
Kerman II [26]	(MCV × MCH × 10)/(RBC × MCHC)	<85	>85
Sirdah [27]	MCV − RBC − (3 × HB)	<27	>27
Ehsani [28]	MCV − (10 × RBC)	<15	>15
Keikhaei [29]	(HB × RDW × 100)/(RBC × RBC × MCHC)	<21	>21
Nishad–Thal index [30]	(0.615 × MCV) + (0.518 × MCH) + (0.446 × RDW)	<59	>59
Wongprachum [31]	(MCV × RDW/RBC) − (10 × HB)	<104	>104
Sirachainan [32]	(1.5 × HB) − (0.05 × MCV)	<972	>972
Sehgal [33]	(MCV × MCV)/RBC	>14	<14
Bordbar [34]	(80 − MCV) × (27 − MCH)	>44.76	<44.76
Hisham [35]	(MCH × RDW)/RBC	<67.0	>67.0
Chandra [36]	(RBC × MCHC × MPV)/(RDW × PQ)	>0.22	<0.22
Matos and Carvalho [37]	(1.91 × RBC) + (0.44 × MCHC)	>23.85	<23.85
Kandhro 1 [38]	(RBC/HTO) + (0.5 × RDW)	<8.2	>8.2
Kandhro 2 [38]	(RDW × 5)/RBC	<16.8	>16.8
CRUISE–Jahangiri [39]	MCHC + (0.603 × RBC) + (0.523 × RDW)	≥42.63	<42.63

HB: hemoglobin; HTO: hematocrit; MCH: mean corpuscular hemoglobin; MCHC: mean corpuscular hemoglobin concentration; MCHD: mean cell hemoglobin density; MCV: mean corpuscular volume; MDHL: mean density of Hb/liter of blood; MPV: mean platelet volume; PQ: platelet count; RBC: red blood cell count; RDW: red cell distribution width.

**Table 2:** Confusion matrix of discriminatory RBC indices.

RBC Index	ΒTT (+)	BTT (−)/(IDA)	Total
England and Fraser I [14]	26 (TP)	1 (FP)	27 (Positives)
	25 (FN)	130 (TN)	155 (Negatives)
	51 (Sick patients)	131 (Healthy patients)	182
Srivastava [15]	40 (TP)	11 (FP)	51 (Positives)
	11 (FN)	120 (TN)	131 (Negatives)
	51 (Sick patients)	131 (Healthy patients)	182
Mentzer [16]	46 (TP)	16 (FP)	62 (Positives)
	5 (FN)	115 (TN)	120 (Negatives)
	51 (Sick patients)	131 (Healthy patients)	182
Shine and Lal [17]	51 (TP)	70 (FP)	121 (Positives)
	0 (FN)	61 (TN)	61 (Negatives)
	51 (Sick patients)	131 (Healthy patients)	182
England and Fraser II [18]	37 (TP)	2 (FP)	39 (Positives)
	14 (FN)	129 (TN)	143 (Negatives)
	51 (Sick patients)	131 (Healthy patients)	182
Bessman [19]	38 (TP)	64 (FP)	102 (Positives)
	13 (FN)	67 (TN)	80 (Negatives)
	51 (Sick patients)	131 (Healthy patients)	182
Ricerca [20]	51 (TP)	69 (FP)	120 (Positives)
	0 (FN)	62 (TN)	62 (Negatives)
	51 (Sick patients)	131 (Healthy patients)	182
Green and King [21]	49 (TP)	5 (FP)	54 (Positives)
	2 (FN)	126 (TN)	128 (Negatives)
	51 (Sick patients)	131 (Healthy patients)	182
Das Gupta [22]	51 (TP)	85 (FP)	136 (Positives)
	0 (FN)	46 (TN)	46 (Negatives)
	51 (Sick patients)	131 (Healthy patients)	182
Jayabose RDW [23]	51 (TP)	21 (FP)	72 (Positives)
	0 (FN)	110 (TN)	110 (Negatives)
	51 (Sick patients)	131 (Healthy patients)	182
Telmissani MCHD [24]	50 (TP)	131 (FP)	181 (Positives)
	1 (FN)	0 (TN)	1 (Negatives)
	51 (Sick patients)	131 (Healthy patients)	182
Telmissani MDHL [24]	34 (TP)	2 (FP)	36 (Positives)
	17 (FN)	129 (TN)	146 (Negatives)
	51 (Sick patients)	131 (Healthy patients)	182
Huber–Herklotz [25]	38 (TP)	6 (FP)	44 (Positives)
	13 (FN)	125 (TN)	138 (Negatives)
	51 (Sick patients)	131 (Healthy patients)	182
Kerman I [26]	48 (TP)	28 (FP)	76 (Positives)
	3 (FN)	103 (TN)	106 (Negatives)
	51 (Sick patients)	131 (Healthy patients)	182
Kerman II [26]	43 (TP)	14 (FP)	57 (Positives)
	8 (FN)	117 (TN)	125 (Negatives)
	51 (Sick patients)	131 (Healthy patients)	182
Sirdah [27]	42 (TP)	2 (FP)	44 (Positives)
	9 (FN)	129 (TN)	138 (Negatives)
	51 (Sick patients)	131 (Healthy patients)	182
Ehsani [28]	46 (TP)	14 (FP)	60 (Positives)
	5 (FN)	117 (TN)	122 (Negatives)
	51 (Sick patients)	131 (Healthy patients)	182
Keikhaei [29]	50 (TP)	11 (FP)	61 (Positives)
	1 (FN)	120 (TN)	121 (Negatives)
	51 (Sick patients)	131 (Healthy patients)	182
Nishad–Thal index [30]	40 (TP)	11 (FP)	51 (Positives)
	11 (FN)	120 (TN)	131 (Negatives)
	51 (Sick patients)	131 (Healthy patients)	182
Wongprachum [31]	51 (TP)	12 (FP)	63 (Positives)
	0 (FN)	119 (TN)	119 (Negatives)
	51 (Sick patients)	131 (Healthy patients)	182
Sirachainan [32]	31 (TP)	6 (FP)	37 (Positives)
	20 (FN)	125 (TN)	145 (Negatives)
	51 (Sick patients)	131 (Healthy patients)	182
Sehgal [33]	50 (TP)	22 (FP)	72 (Positives)
	1 (FN)	109 (TN)	110 (Negatives)
	51 (Sick patients)	131 (Healthy patients)	182
Bordbar [34]	50 (TP)	39 (FP)	89 (Positives)
	1 (FN)	92 (TN)	93 (Negatives)
	51 (Sick patients)	131 (Healthy patients)	182
Hisham [35]	51 (TP)	23 (FP)	74 (Positives)
	0 (FN)	108 (TN)	108 (Negatives)
	51 (Sick patients)	131 (Healthy patients)	182
Chandra [36]	51 (TP)	84 (FP)	135 (Positives)
	0 (FN)	47 (TN)	47 (Negatives)
	51 (Sick patients)	131 (Healthy patients)	182
Matos and Carvalho [37]	46 (TP)	4 (FP)	50 (Positives)
	5 (FN)	127 (TN)	132 (Negatives)
	51 (Sick patients)	131 (Healthy patients)	182
Kandhro 1 [38]	50 (TP)	98 (FP)	148 (Positives)
	1 (FN)	33 (TN)	34 (Negatives)
	51 (Sick patients)	131 (Healthy patients)	182
Kandhro 2 [38]	51 (TP)	74 (FP)	125 (Positives)
	0 (FN)	57 (TN)	57 (Negatives)
	51 (Sick patients)	131 (Healthy patients)	182
CRUISE–Jahangiri [39]	27 (TP)	21 (FP)	48 (Positives)
	24 (FN)	110 (TN)	134 (Negatives)
	51 (Sick patients)	131 (Healthy patients)	182

BTT: β-thalassemia trait; FN: false negative; FP: false positive; IDA: iron deficiency anemia; MCHD: mean cell hemoglobin density; MDHL: mean density of Hb/liter of blood; RBC: red blood cell count; RDW: red-cell distribution width; TN: true negative; TP: rue positive.

**Table 3:** Likelihood ratio value ranges and their impacts on clinical utility.

PLR	NLR	Utility
10	<0.1	Highly relevant
5–10	0.1–0.2	Good
2–5	0.5–0.2	Regular
<2	>0.5	Bad

**Table 4:** Diagnostic performance of discriminatory RBC indices.

RBC Index	Se (CI 95%)	Sp (CI 95%)	PPV (CI 95%)	NPV (CI 95%)	PLR (CI 95%)	NLR (CI 95%)	Accuracy (CI 95%)	Youden’s Index
England and Fraser I [14]	51.0% (36.6–65.3)	99.2% (95.8–100.0)	96.3% (78.4–99.5)	83.9% (79.7–87.3)	66.78 (9.30–479.4)	0.49 (0.37–0.65)	85.7% (79.8–90.5)	0.502
Srivastava [15]	78.4% (64.7–88.7)	91.6% (85.5–95.7)	78.4% (67.0–86.7)	91.6% (86.6–94.9)	9.34 (5.21–16.74)	0.24 (0.14–0.40)	87.9% (82.3–92.3)	0.700
Mentzer [16]	90.2% (78.6–96.7)	87.8% (80.9–92.9)	74.2% (64.3–82.1)	95.8% (90.9–98.2)	7.38 (4.63–11.79)	0.11 (0.05–0.26)	88.5% (82.9–92.7)	0.780
Shine and Lal [17]	100.0% (93.0–100.0)	46.6% (37.8–55.5)	42.1% (38.3–46.1)	100.0% (94.1–100.0)	1.87 (1.59–2.20)	0.00 (NA)	61.5% (54.1–68.6)	0.466
England and Fraser II [18]	72.5% (58.3–84.1)	98.5% (94.6–99.8)	94.9% (82.2–98.7)	90.2% (85.5–93.5)	47.52 (11.89–189.9)	0.28 (0.18–0.44)	91.2% (86.1–94.9)	0.710
Bessman [19]	74.5% (60.4–85.7)	51.1% (42.3–60.0)	37.3% (31.9–43.0)	83.8% (75.8–89.5)	1.53 (1.20–1.93)	0.50 (NA)	57.7% (50.2–65.0)	0.257
Ricerca [20]	100.0% (93.0–100.0)	47.3% (38.6–56.2)	42.5% (38.6–46.5)	100.0% (94.2–100.0)	1.90 (1.61–2.23)	0.00 (NA)	62.1% (54.6–69.2)	0.473
Green and King [21]	96.1% (86.5–99.5)	96.2% (91.3–98.8)	90.7% (80.6–95.9)	98.4% (94.2–99.6)	25.17 (10.64–59.57)	0.04 (0.01–0.16)	96.2% (92.2–98.4)	0.923
Das Gupta [22]	100.0% (93.0–100.0)	35.1% (27.0–43.9)	37.5% (34.6–40.5)	100.0% (92.3–100.0)	1.54 (1.36–1.75)	0.00 (NA)	53.3% (45.8–60.7)	0.351
Jayabose RDW [23]	100.0% (93.0–100.0)	84.0% (76.6–89.8)	70.8% (62.1–78.2)	100.0% (96.7–100.0)	6.24 (4.22–9.23)	0.00 (NA)	88.5% (82.9–92.7)	0.840
Telmissani MCHD [24]	98.0% (89.6–100.0)	0.0% (0.0–2.8)	27.6% (26.9–28.4)	0.0% (NA)	0.98 (0.94–1.02)	NA	27.5% (21.1–34.6)	−0.020
Telmissani MDHL [24]	66.7% (52.1–79.2)	98.5% (94.6–99.8)	94.4% (80.9–98.6)	88.4% (83.7–91.8)	43.67 (10.89–175.1)	0.34 (0.23–0.50)	89.6% (84.2–93.6)	0.651
Huber–Herklotz [25]	74.5% (60.4–85.7)	95.4% (90.3–98.3)	86.4% (74.0–93.4)	90.6% (85.7–93.9)	16.27 (7.32–36.13)	0.27 (0.17–0.43)	89.6% (84.2–93.6)	0.699
Kerman I [26]	94.1% (83.8–98.8)	78.6% (70.6–85.3)	63.2% (55.1–70.6)	97.2% (91.9–99.0)	4.40 (3.15–6.16)	0.07 (0.02–0.23)	83.0% (76.7–88.1)	0.727
Kerman II [26]	84.3% (71.4–93.0)	89.3% (82.7–94.0)	75.4% (64.9–83.6)	93.6% (88.5–96.5)	7.89 (4.74–13.13)	0.18 (0.09–0.33)	87.9% (82.3–92.3)	0.736
Sirdah [27]	82.4% (69.1–91.6)	98.5% (94.6–99.8)	95.5% (84.1–98.8)	93.5% (88.8–96.3)	53.94 (13.55–214.7)	0.18 (0.10–0.32)	94.0% (89.4–96.9)	0.808
Ehsani [28]	90.2% (78.6–94.7)	89.3% (82.7–94.0)	76.7% (66.5–84.5)	95.9% (91.0–98.2)	8.44 (5.10–13.96)	0.11 (0.05–0.25)	89.6% (84.2–93.6)	0.795
Keikhaei [29]	98.0% (89.6–100.0)	91.6% (85.5–95.7)	82.0% (72.1–88.9)	99.2% (94.5–99.9)	11.68 (6.62–20.58)	0.02 (0.00–0.15)	93.4% (88.8–96.6)	0.896
Nishad–Thal index [30]	78.4% (64.7–88.7)	91.6% (85.5–95.7)	78.4% (67.0–86.7)	91.6% (86.6–94.9)	9.34 (5.21–16.74)	0.24 (0.14–0.40)	87.9% (82.3–92.3)	0.700
Wongprachum [31]	100.0% (93.0–100.0)	90.8% (84.6–95.2)	81.0% (71.3–87.9)	100.0% (97.0–100.0)	10.92 (6.37–18.72)	0.00 (NA)	93.4% (88.8–96.6)	0.908
Sirachainan [32]	60.8% (46.1–74.2)	95.4% (90.3–98.3)	83.8% (69.6–92.1)	86.2% (81.6–89.8)	13.27 (5.89–29.90)	0.41 (0.29–0.58)	85.7% (79.8–90.5)	0.562
Sehgal [33]	98.0% (89.6–100.0)	83.2% (75.7–89.2)	69.4% (60.8–76.9)	99.1% (94.0–99.9)	5.84 (3.98–8.56)	0.02 (0.00–0.16)	87.4% (81.6–91.8)	0.812
Bordbar [34]	98.0% (89.6–100.0)	70.2% (61.6–77.9)	56.2% (49.6–62.6)	98.9% (92.9–99.8)	3.29 (2.52–4.30)	0.03 (0.00–0.20)	78.0% (71.3–83.8)	0.683
Hisham [35]	100.0% (93.0–100.0)	82.4% (74.8–88.5)	68.9% (60.5–76.3)	100.0% (96.6–100.0)	5.70 (3.93–8.25)	0.00 (NA)	87.4% (81.6–91.8)	0.824
Chandra [36]	100.0% (93.0–100.0)	35.9% (27.7–44.7)	37.8% (34.8–40.8)	100.0% (92.5–100.0)	1.56 (1.37–1.77)	0.00 (NA)	53.8% (46.3–61.3)	0.359
Matos and Carvalho [37]	90.2% (78.6–96.7)	96.9% (92.4–99.2)	92.0% (81.4–96.8)	96.2% (91.7–98.3)	29.54 (11.21–77.86)	0.10 (0.04–0.23)	95.1% (90.8–97.7)	0.871
Kandhro 1 [38]	98.0% (89.6–100.0)	25.2% (18.0–33.5)	33.8% (31.4–36.2)	97.1% (82.3–99.6)	1.31 (1.18–1.46)	0.08 (0.01–0.55)	45.6% (38.2–53.1)	0.232
Kandhro 2 [38]	100.0% (93.0–100.0)	43.5% (34.9–52.5)	40.8% (37.2–44.5)	100.0% (93.7–100.0)	1.77 (1.52–2.06)	0.00 (NA)	59.3% (51.8–66.6)	0.435
CRUISE–Jahangiri [39]	52.9% (38.5–67.1)	84.0% (76.6–89.8)	56.3% (44.6–67.3)	82.1% (77.2–86.1)	3.30 (2.06–5.28)	0.56 (0.41–0.76)	75.3% (68.4–81.4)	0.369

CI: confidence interval; MCHD: mean cell hemoglobin density; MDHL: mean density of Hb/liter of blood; NA: not applicable; NLR: negative likelihood ratio; NPV: negative predictive value; PLR: positive likelihood ratio; PPV: positive predictive value; RBC: red blood cell count; RDW: red-cell distribution width; Se: sensitivity; Sp: specificity.

**Table 5:** Complete blood count results in the β-thalassemia trait and iron deficiency anemia groups.

	BTT (*n* = 51)		IDA (*n* = 131)		*p*
	Mean (±SD)	Range	Mean (±SD)	Range	
RBC (×10^6^/mm^3^)	5.92 ± 0.61	5.00–7.41	4.56 ± 0.49	3.19–5.99	<0.0001 *
HB (g/dL)	12.14 ± 1.21	10.0–15.3	10.69 ± 0.92	9.0–12.9	<0.0001 *
HTO (%)	38.47 ± 3.87	31.9–49.3	34.55 ± 2.54	29.0–41.6	<0.0001 *
MCV (fL)	64.98 ± 3.17	57.4–70.9	76.35 ± 7.09	58.6–92.8	<0.0001 *
MCH (pg)	20.55 ± 1.08	17.8–22.6	23.67 ± 2.81	18.1–30.7	<0.0001 *
MCHC (%)	31.57 ± 0.69	30.3–33.2	30.94 ± 1.14	26.5–33.1	0.0003 *
PQ (×10^3^/mm^3^)	292.2 ± 78.6	130–493	345.7 ± 85.1	158–624	0.0001 *
MPV (fL)	9.19 ± 0.84	7.7–12.3	8.86 ± 0.91	6.3–11.2	0.0261 *
RDW (%)	14.52 ± 0.82	13.0–18.0	15.32 ± 2.18	11.8–23.5	0.0117 *
Basophilic stippling	90.2% (46/51)		0.0% (0/131)		<0.0001 *

BTT: β-thalassemia trait; HB: hemoglobin; HTO: hematocrit; IDA: iron deficiency anemia; MCH: mean corpuscular hemoglobin; MCHC: mean corpuscular hemoglobin concentration; MCV: mean corpuscular volume; MPV: mean platelet volume; PQ: platelet count; RBC: red blood cell count; RDW: red-cell distribution width; SD: standard deviation. * Statistically significant differences (*p* < 0.05).

## Data Availability

The original contributions presented in this study are available upon request.

## Supplementary Materials

The following supporting information can be downloaded at https://www.mdpi.com/article/10.3390/diagnostics14212353/s1: Table S1: Online RBC index calculator.
